# Gazing into smoldering volcanoes: precision cardiac imaging

**DOI:** 10.4155/fsoa-2018-0009

**Published:** 2018-04-06

**Authors:** Alastair J Moss, Stuart Hutchison, Marc R Dweck

**Affiliations:** 1 BHF Centre for Cardiovascular Science, University of Edinburgh, Edinburgh, UK

**Keywords:** cardiovascular, medical imaging, myocardial infarction, personalized medicine

“The good physician treats the disease; the great physician treats the patient who has the disease” – William Osler.

“To enable a new era of medicine through research, technology and policies that empower patients, researchers and providers to work together toward development of individualized care” – mission statement of the Precision Medicine Initiative.

Clinicians routinely make intuitive decisions that aim to ‘personalize’ an individual's treatment. This is far from an exact science, and often results in clinicians oversimplifying the complexity of the underlying pathophysiology. In a time-pressured clinical environment, clinicians use intuitive decision-making to make fast and impulsive judgments that often attribute a patient's complaint to a single common phenotype leading to a ‘one-size-fits-all’ approach to treatment. This population-approach to myocardial infarction has yielded better use of evidence-based therapies and promotion of lifestyle modifications to dramatically reduce early deaths from myocardial infarction over the past two decades. However, many patients fail to benefit from the widely advocated successes of a ‘one-size-fits-all’ approach either by sustaining harm from treatment or by remaining at increased risk of recurrent cardiovascular events despite optimal therapeutic interventions [1]. Of note, the main risk of a complication from a myocardial infarction is no longer in the early phase during the initial hospital admission, but after discharge from hospital when one in five patients may suffer a recurrent cardiac event within 5 years. After an ST elevation myocardial infarction, 68% of cardiovascular deaths occur following discharge from hospital and this rises to 86% in cases of non-ST elevation myocardial infarction. A particular concern is the failure for validated risk scores to accurately predict the risk of recurrent myocardial infarction. The challenge faced by cardiologists is to maintain these improvements in early cardiovascular outcomes after myocardial infarction and translate them into sustained longer-term benefits.

## Residual risk of recurrent events is a clinical priority

To influence long-term outcomes, a more considered and logical approach is required. Precision medicine requires taking account of the heterogeneity of disease and an individual's variable response to treatment. A detailed insight into the biological continuum of disease will help unravel the complexity of why some individuals gain significant benefits from interventions whereas others do not. Whereas current strategies in coronary artery disease have focused on population-derived evidence-based therapies, precision medicine seeks to understand an individual's unique biological network using ‘omics’ (epigenetics, genetics, transcriptomics, proteomics and metabolomics) to stratify treatment. The life-threatening complications that afflict individuals following an acute myocardial infarction despite optimal medical therapy are commonly referred to as the concept of residual risk.

## A ‘one-size-fits-all’ approach is not cost-effective in current healthcare systems

The multinational INTERHEART study demonstrated that nine modifiable risk factors account for over 90% of an individual's future cardiovascular risk [2]. Suboptimal control of cardiovascular risk factors, particularly the underdiagnosis and failure to tailor therapy for individuals with elevated low-density lipoprotein cholesterol, hypertension and Type 2 diabetes mellitus may be potential explanations for the residual risk observed in clinical practice [3]. Preventing myocardial infarctions in this group has required substantial investment from researchers in the field of cardiovascular disease to attain marginal gains with unselected use of adjuvant therapy. Two recent randomized placebo-controlled trials have demonstrated that the residual risk of all-cause mortality following myocardial infarction and the initiation of optimal medical therapy is 0.80–1.43 per 100 patient years [4,5]. Novel monoclonal antibodies directed against proprotein convertase subtilisin-kexin type 9 and IL-β hold promise for attenuating the residual risk of recurrent myocardial infarction, but to date, they have not resulted in a significant reduction in cardiovascular death or all-cause mortality. A limitation of this data in translating these trials to real-world practice is that the biomarkers used for inclusion in these trials (low-density lipoprotein cholesterol or high sensitivity C-reactive protein) are weak at stratifying future cardiovascular risk of myocardial infarction. Indeed, high-sensitivity C-reactive protein failed to identify 41% of first presentation ST elevation myocardial infarctions in a multiethnic population [6]. The high cost of monoclonal antibodies in the current financial climate will prohibit the widespread use of these therapies unless a more accurate method of refining residual risk can be attained. To achieve this goal, researchers are investigating how novel imaging techniques add precision to the phenotyping of coronary artery disease.

## Cardiovascular imaging improves the stratification of treatment

While cardiovascular imaging techniques are not a therapeutic tool in themselves, the information obtained can influence downstream management by confirming a diagnosis and better selecting the prescription of treatment to reduce the complications of cardiovascular disease. Despite the ubiquitous use of imaging in cardiovascular medicine, until recently no randomized clinical trials have evaluated whether incorporating an imaging technique into a treatment pathway actually improves the care of patients. The first trial to do so was the SCOT-HEART trial published in 2015, which sought to ascertain the impact of computed tomography coronary angiography in the diagnosis and management of patients with stable chest pain due to suspected coronary artery disease in an outpatient clinic setting [7]. It found that when computed tomography coronary angiography was added to standard care, it clarified the diagnostic certainty of coronary artery disease and after the implementation of preventative therapy halved the number of fatal and nonfatal myocardial infarctions over 3 years [7,8].

This trial exemplifies that even in a clinical setting where evidence-based practice is already in place, significant improvements can be achieved by adopting a more precise approach to the classification of coronary artery disease.

## Molecular imaging of coronary artery disease

Attention is now focused on identifying the pathophysiology of coronary artery disease *in vivo*. Advances in hybrid cardiovascular imaging with the emergence of positron-emission tomography–computed tomography have allowed investigators to visualize disease activity at the molecular level within specific vascular territories. The ability to directly observe a pathophysiological process *in vivo* using molecular imaging has transformed oncological practice where precision medicine plays an integral role in the detection of malignancy and evaluation of response to treatment. Translation of this imaging technique into the field of coronary imaging has been facilitated with technological improvements in scanner resolution and ECG-gated acquisition. Precision medicine using positron-emission tomography is a different concept from an ‘omics’ approach to categorizing disease. A strength of recent cardiovascular genetic studies is the observation that genetic polymorphisms significantly influence the pharmacodynamics of drug therapy. The inheritability of these polymorphisms therefore allows for pre-emptive screening to mitigate against adverse responses to drug therapy. However, partly due to the complex and multifactorial nature of coronary atherosclerosis, candidate gene association studies have delivered widely conflicting results for predicting risk of recurrent myocardial infarction. For instance, whereas genetic variants associated with Chromosome 9p21 were strongly associated with the first myocardial infarction, they did not stratify individuals at risk of further cardiac events [9]. Once the natural course of the disease is modified by therapeutic intervention, more responsive markers of disease activity may be required. This is the rationale for using positron emission tomographic imaging to identify *in vivo* molecular targets of active atherosclerosis.

Molecular targets of inflammation in the coronary and carotid arteries can be detected using 2-[18F]-Fluoro-2-deoxy-D-glucose imaging [10]. This molecular tracer has been used as an imaging biomarker in early phase clinical trials with the accurate prediction of subsequent clinical response to atorvastatin [11], pioglitazone [12], dalcetrapib [13], losmapimod [14], oxidized LDL inhibitor [15] and lipoxygenase inhibitor [16]. Considerable interest surrounds the recently reported use of 68Ga-DOTATATE, which more specifically identifies proinflammatory M1 macrophages by binding to the somatostatin subtype 2 receptor [17]. As the number of molecular imaging markers continues to grow, our understanding of the mechanisms governing atherosclerosis also continues to evolve. In this regard, microcalcification has been implicated in the role of coronary artery plaque rupture and can be clearly visualized using 18F-fluoride [18]. 18F-fluoride binds to exposed hydroxyapatite and enables detection of microcalcification below the resolution of conventional cardiovascular imaging techniques such as computed tomography. It is posited that nanocrystalline deposition of hydroxyapatite occurs in response to the inflammatory and hypoxic milieu generated in necrotic cores of apoptotic macrophages. These are regions in coronary arteries of ‘smoldering’ activity highly associated with plaque rupture and ‘eruption’ of prothrombogenic material.

Considerable interest now centers on identifying these areas of activity in patients with myocardial infarction to evaluate whether secondary prevention can be better stratified to those at high-risk of recurrent cardiovascular events. This is the key research question for the ongoing prospective observational PRE18FFIR study (NCT02278211). The ability to provide a detailed phenotype of the activity and progression of pathophysiology *in vivo* using molecular imaging is an enticing way of delivering precision cardiovascular medicine.
